# A U-shaped association between the LDL-cholesterol to HDL-cholesterol ratio and all-cause mortality in elderly hypertensive patients: a prospective cohort study

**DOI:** 10.1186/s12944-020-01413-5

**Published:** 2020-11-12

**Authors:** Yu Yu, Minghui Li, Xiao Huang, Wei Zhou, Tao Wang, Lingjuan Zhu, Congcong Ding, Yu Tao, Huihui Bao, Xiaoshu Cheng

**Affiliations:** 1 Department of Cardiovascular Medicine, the Second Affiliated Hospital of Nanchang University, No. 1 Minde Road, Nanchang, 330006 Jiangxi China; 2 Center for Prevention and Treatment of Cardiovascular Diseases, the Second Affiliated Hospital of Nanchang University, Nanchang, Jiangxi China

**Keywords:** Low-density lipoprotein cholesterol to high-density lipoprotein cholesterol ratio, U-shaped curve, Mortality, Hypertension, Low-density lipoprotein cholesterol, High-density lipoprotein cholesterol, Cohort study

## Abstract

**Background:**

The low-density lipoprotein cholesterol/high-density lipoprotein- cholesterol (LDL-C/HDL-C) ratio is an excellent predictor of cardiovascular disease (CVD). However, previous studies linking the LDL-C/HDL-C ratio to mortality have yielded inconsistent results and been limited by short follow-up periods. Therefore, the aim of the present study was to determine whether the LDL-C/HDL-C ratio could be an effective predictor of all-cause mortality in elderly hypertensive patients.

**Methods:**

A total of 6941 hypertensive patients aged 65 years or older who were not treated with lipid-lowering drugs were selected from the Chinese Hypertension Registry for analysis. The endpoint of the study was all-cause mortality. The relationship between the LDL-C/HDL-C ratio and all-cause mortality was determined using multivariate Cox proportional hazards regression, smoothing curve fitting (penalized spline method), subgroup analysis and Kaplan–Meier survival curve analysis.

**Results:**

During a median follow-up of 1.72 years, 157 all-cause deaths occurred. A U-shaped association was found between the LDL-C/HDL-C ratio and all-cause mortality. Patients were divided according to the quintiles of the LDL-C/HDL-C ratio. Compared to the reference group (Q3: 1.67–2.10), patients with both lower (Q1 and Q2) and higher (Q4 and Q5) LDL-C/HDL-C ratios had higher all-cause mortality (< 1.67: HR 1.81, 95% CI: 1.08–3.03; ≥2.10: HR 2.00, 95% CI: 1.18–3.39). Compared with the lower and higher LDL-C/HDL-C ratio groups, patients with LDL-C/HDL-C ratios of 1.67–2.10 had a significantly higher survival probability (log-rank *P* = 0.038).

**Conclusions:**

The results suggest that there is a U-shaped association between the LDL-C/HDL-C ratio and all-cause mortality. Both lower and higher LDL-C/HDL-C ratios were associated with increased all-cause mortality in elderly hypertensive patients.

**Supplementary Information:**

The online version contains supplementary material available at 10.1186/s12944-020-01413-5.

## Background

The leading causes of death worldwide include stroke and cardiovascular disease (CVD), both of which are associated with higher levels of low-density lipoprotein cholesterol (LDL-C) and lower levels of high-density lipoprotein cholesterol (HDL-C) [1, 2]. The LDL-C/HDL-C ratio, which is a new marker, is calculated by dividing LDL-C by HDL-C. Enomoto M et al. [3] followed 1920 Japanese individuals for 8 years, and found that the LDL-C/HDL-C ratio had greater predictive value for carotid intima-media thickness than LDL-C or HDL-C alone. Ridker PM et al. [4] followed 15,632 US women for 10 years and found that compared with LDL-C or HDL-C, the LDL-C/HDL-C ratio had a stronger relationship with the CVD composite endpoint, which included myocardial infarction, stroke, coronary revascularization, and cardiovascular-related death. CVD and death are closely related, and the LDL-C/HDL-C ratio should also be suitable for predicting death. However, previous studies on the LDL-C/HDL-C ratio have drawn inconsistent conclusions. Some studies have suggested that the LDL-C/HDL-C ratio is positively associated with CVD [5–8], while others have found a negative correlation between the LDL-C/HDL-C ratio and all-cause mortality [9, 10]. The reasons for these inconsistencies can be attributed to differences in the study population, follow-up, and study endpoints. Furthermore, the above studies proposed different ranges of the LDL-C/HDL-C ratio that were associated with the lowest risk of CVD or all-cause mortality. These factors prevent the LDL-C/HDL-C ratio from being a valuable predictor of all-cause mortality in clinical practice.

In addition, there have been some limitations of the studies on the relationship between LDL-C/HDL-C ratio and all-cause mortality, including small sample sizes, short follow-up durations, and the varying disease severity; therefore, the relationship between the LDL-C/HDL-C ratio and all-cause mortality remains unclear. Notably, elderly hypertensive patients (defined as hypertensive patients over the age of 65 years) have higher mortality than the rest of the population [11]. Therefore, it is necessary for researchers to identify a clinically valuable predictor of all-cause mortality in elderly patients with hypertension.

In an effort to address the significant gaps in knowledge, the present study was performed to investigate the potential relationship of the LDL-C/HDL-C ratio with all-cause mortality, and to examine the optimal range of the LDL-C/HDL-C ratio in the hypertensive population aged 65 years or older, using data from the China Hypertension Registry Study.

## Methods

### Study design and participants

The study data were drawn from the China Hypertension Registry Study (http://www.chictr.org.cn/, No: ChiCTR1800017274). Details of the methodology, and primary objectives of this study had been described elsewhere [12]. The inclusion and exclusion criteria for the study are described in detail in Table S1. In short, this study is an observational, real-world investigation of hypertension. Hypertension was defined by a systolic blood pressure (SBP) ≥140 mmHg and/or diastolic BP (DBP) ≥90 mmHg, self-reported history of hypertension, or the use of antihypertensive drug(s) at baseline [13]. From March 2018 to August 2018, 14,268 patients with hypertension were recruited in Wuyuan, Jiangxi Province, China, and the treatment, related risk factors and prognosis of patients with hypertension were evaluated. In our study, we selected 6941 hypertensive patients aged 65 years or older who were not treated with lipid-lowering drugs from the China Hypertension Registry Study. The study protocol was approved by the Ethics Committee of the Anhui Medical University Biomedical Institute (No. CH1059), and all participants signed informed consent.

### Data collection and outcome definition

All study participants had fasting, venous blood samples collected by trained study staff during the baseline data collection period. Total cholesterol (TC, mmol/L), triglycerides (TG, mmol/L), LDL-C (mmol/L), HDL-C (mmol/L), serum uric acid (SUA, μmol/L), the estimated glomerular filtration rate (eGFR, ml/min/1.73m^2^), homocysteine (Hcy, μmol/L), fasting blood glucose (FBG, mmol/L) and albumin (g/L) were measured by an automatic clinical analyzer (Beckman Coulter, USA) in Biaojia Biotechnology Laboratory, Shenzhen, China [14]. The LDL-C/HDL-C ratio was calculated by dividing LDL-C by HDL-C. In addition to the abovementioned laboratory indicators, other covariates in the study included age (years), sex (male or female), body mass index (BMI, kg/m^2^), smoking status (never, former and current), alcohol consumption, history of disease (including stroke, CVD and diabetes), systolic/diastolic blood pressure (mmHg) and drug history (including antihypertensive drugs, antiplatelet drugs and glucose-lowering drugs).

The outcome of our study was all-cause mortality from 31 August 2018 to 31 March 2020. Causes of death included stroke, CVD, tumor respiratory diseases, and other reasons. All-cause mortality was ascertained from the Local Healthcare Security Administration, Centers for Disease Control and Prevention, and hospitals.

### Statistical analysis

Study population characteristics at baseline are presented stratified by the quintiles of the LDL-C/HDL-C ratio. Data are presented as the means ± SDs or proportions. The Mann-Whitney test was used to identify significant differences between the two groups. Multiple Cox proportional hazards regression analysis was used to analyze the relationship between the LDL-C/HDL-C ratio and all-cause mortality, and the results are presented as hazard ratios (HRs) and 95% confidence intervals (CIs). The adjusted variables were selected based on their clinical importance, statistical significance in the univariable analysis, and potential confounding, indicated by estimates that individually changed by at least 10%. Fully adjusted smoothing curve fitting (penalized spline method) visually showed the relationship between the LDL-C/HDL-C ratio and all-cause mortality. Stratified analyses were conducted, with stratification by sex, BMI, stroke, CVD, diabetes and eGFR, to identify potential subgroups in which there was a significant association between the LDL-C/HDL-C ratio and all-cause mortality. Survival was estimated by the Kaplan-Meier method, and any differences in survival were evaluated with stratified log-rank tests.

All data analysis and form generation were performed using the statistical package R (http://www.R-project.org, The R Foundation) and Empower (R) (www.empowerstats.com; X&Y Solutions, Inc., Boston, MA). When a two-tailed *P* was < 0.05, we had sufficient reason to believe that the results were statistically significant.

## Results

### Baseline characteristics of the study participants

A total of 6941 hypertensive patients aged 65 years or older who were not treated with lipid-lowering drugs were selected for the final data analysis (mean age: 71.20 ± 5.30; male: 48.18%). The distribution of the baseline population characteristics according to the baseline LDL-C/HDL-C ratio quintiles is described in Table 1. Compared with the lower groups (Q1 and Q2), the higher groups (Q4 and Q5) had higher values of DBP, TC, TG, LDL-C, FBG, albumin, and SUA; were more likely to have stroke and diabetes; and were more likely to take antihypertensive drugs and glucose-lowering drugs. In contrast, the higher groups (Q4 and Q5) were younger; less likely to be male; less likely to smoke or consume alcohol; and had lower values of HDL-C and the eGFR (all *P* < 0.05).

**Table 1:** Baseline Characteristics of the Cohort Per Quintiles of the LDL-C/HDL-C ratio

Characteristics*	Quintiles of the LDL-C/HDL-C ratio					*P* value
	Q1 (< 1.16)	Q2 (1.16–1.67)	Q3 (1.67–2.10)	Q4 (2.10–2.79)	Q5 (≥2.79)	
N	1387	1389	1389	1387	1389	
Demographics						
Age, years	71.75 ± 5.51	71.67 ± 5.59	71.10 ± 5.36	70.78 ± 5.06^cf^	70.69 ± 4.89^dg^	< 0.001
Male, %	836 (60.27)	691 (49.75) ^a^	629 (45.28) ^b^	581 (41.89) ^cf^	607 (43.70) ^d^	< 0.001
BMI, kg/m^2^	20.85 ± 4.95	21.91 ± 3.26^a^	22.77 ± 3.07^be^	23.56 ± 3.25^cfh^	24.15 ± 3.18^dgij^	< 0.001
Smoking, %						< 0.001
Never	638 (46.00)	732 (52.74)	778 (56.01)	788 (56.85)	768 (55.33)	
Former	263 (18.96)	258 (18.59)	283 (20.37)	260 (18.76)	259 (18.66)	
Current	486 (35.04)	398 (28.67) ^a^	328 (23.61) ^b^	338 (24.39) ^c^	361 (26.01) ^d^	
Alcohol consumption, %	473 (34.10)	297 (21.40) ^a^	241 (17.35) ^b^	222 (16.02) ^cf^	199 (14.34) ^dg^	< 0.001
History of disease, %						
Stroke	74 (5.34)	78 (5.62)	90 (6.48)	90 (6.49)	130 (9.36) ^d^	0.001
CVD	75 (5.41)	95 (6.84)	86 (6.19)	89 (6.42)	89 (6.41)	0.620
Diabetes	158 (11.39)	180 (12.96)	206 (14.83)	274 (19.75) ^cf^	354 (25.49) ^dgi^	< 0.001
Blood pressure						
Systolic BP, mmHg	149.30 ± 18.83	149.53 ± 18.69	150.66 ± 17.87	150.47 ± 18.18	150.33 ± 18.47	0.199
Diastolic BP, mmHg	84.88 ± 10.86	85.22 ± 10.71	85.77 ± 10.22	85.32 ± 10.07	86.04 ± 10.07	0.030
Lipids						
TC, mmol/L	4.68 ± 0.95	4.95 ± 0.97 ^a^	5.15 ± 1.03 ^be^	5.37 ± 1.07 ^cfh^	5.68 ± 1.19 ^dgij^	< 0.001
TG, mmol/L	0.98 ± 0.44	1.24 ± 0.61 ^a^	1.47 ± 0.72 ^be^	1.89 ± 1.03 ^cfh^	2.37 ± 1.34 ^dgij^	< 0.001
LDL-C, mmol/L	2.21 ± 0.53	2.68 ± 0.55 ^a^	2.98 ± 0.62 ^be^	3.24 ± 0.67 ^cfh^	3.64 ± 0.78 ^dgij^	< 0.001
HDL-C, mmol/L	2.03 ± 0.46	1.74 ± 0.36 ^a^	1.59 ± 0.33 ^be^	1.45 ± 0.30 ^cfh^	1.27 ± 0.27 ^dgij^	< 0.001
LDL-C/HDL-C ratio	1.10 ± 0.20	1.54 ± 0.10 ^a^	1.88 ± 0.10 ^be^	2.24 ± 0.11 ^cfh^	2.89 ± 0.41 ^dgij^	< 0.001
Other plasma parameters						
Hcy, μmol/L	19.10 ± 11.19	19.32 ± 11.28	18.91 ± 11.10	19.18 ± 11.94	19.72 ± 12.54	0.439
FBG, mmol/L	5.90 ± 1.23	6.00 ± 1.35	6.06 ± 1.64	6.19 ± 1.46 ^cf^	6.43 ± 1.81 ^dgij^	< 0.001
Albumin, g/L	45.57 ± 4.27	45.83 ± 4.06	45.90 ± 3.98	46.18 ± 3.89 ^c^	45.98 ± 3.96	0.002
SUA, μmol/L	413.30 ± 123.79	411.09 ± 114.47	415.57 ± 118.22	429.92 ± 122.14 ^cf^	450.71 ± 120.26 ^dgij^	< 0.001
eGFR, ml/min/1.73m^2^	81.52 ± 19.56	81.51 ± 18.15	81.46 ± 19.33	80.44 ± 19.26	78.26 ± 20.21 ^dgi^	< 0.001
Medication use, %						
Antihypertensive drugs	874 (63.01)	897 (64.63)	959 (69.04)	948 (68.40)	983 (70.82) ^d^	< 0.001
Antiplatelet drugs	34 (2.45)	33 (2.38)	38 (2.74)	43 (3.10)	39 (2.81)	0.773
Glucose-lowering drugs	34 (2.45)	47 (3.38)	56 (4.03)	85 (6.13) ^c^	100 (7.20) ^dg^	< 0.001

Abbreviations: *BMI* body mass index; *CVD* cardiovascular disease; *TC* total cholesterol; *TG* triglyceride; *LDL-C* low density lipoprotein cholesterol; *HDL-C* high density lipoprotein cholesterol; *SUA* serum uric acid; *eGFR* estimated glomerular filtration rate; *Hcy* homocysteine; *FBG* fasting blood glucose

*Data are presented as number (%) or mean ± standard deviation

^a^ indicates a significant difference between Q2 and Q1, *P* < 0.001; ^b^ indicates a significant difference between Q3 and Q1, *P* < 0.001; ^c^ indicates a significant difference between Q4 and Q1, *P* < 0.001; ^d^ indicates a significant difference between Q5 and Q1, *P* < 0.001; ^e^ indicates a significant difference between Q3 and Q2, *P* < 0.001; ^f^ indicates a significant difference between Q4 and Q2, *P* < 0.001; ^g^ indicates a significant difference between Q5 and Q2, *P* < 0.001; ^h^ indicates a significant difference between Q4 and Q3, *P* < 0.001; ^i^ indicates a significant difference between Q5 and Q3, *P* < 0.001; ^j^ indicates a significant difference between Q5 and Q4, *P* < 0.001

### U-shaped relationship between LDL-C/HDL-C ratio and all-cause mortality

**Fig. 1:**
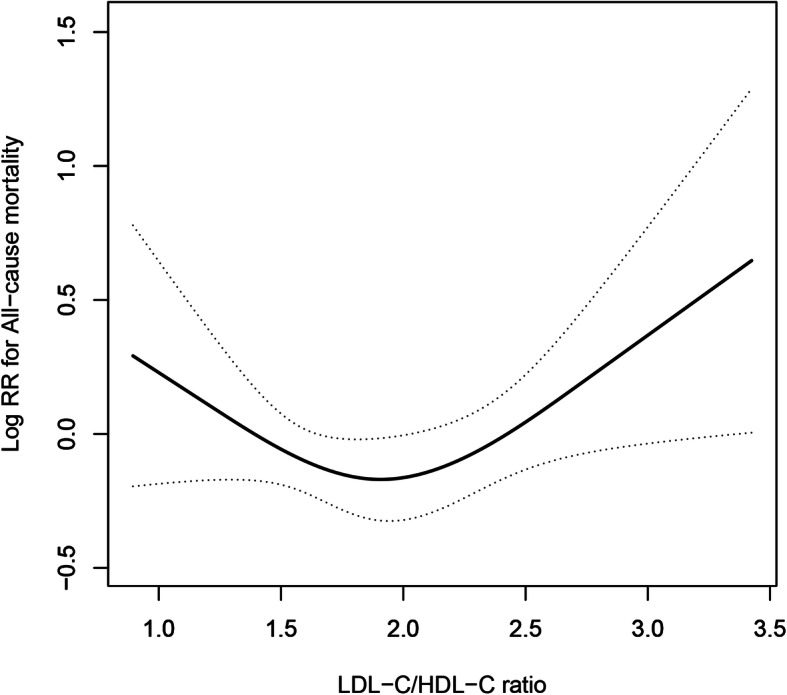
Dose-response relationship between the LDL-C/HDL-C and the probability of all-cause mortality. The smooth curve fitting presented a U-shaped relationship between the LDL-C/HDL-C ratio and all-cause mortality. Adjusted for age, sex, BMI, SBP, DBP, TG, Hcy, FBG, SUA, eGFR, smoking, alcohol consumption, diabetes, stroke, CVD and anti-hypertensive drugs

In the present study, the average follow-up duration was 1.72 years, and a total of 157 all-cause deaths (2.26%) occurred during the follow-up period. Table 2 shows the HR and 95% CI values of the relationship between the LDL-C/HDL-C ratio and all-cause mortality in the crude model, model 1 (partially adjusted) and model 2 (fully adjusted). Multiple Cox proportional hazard regression analysis was used to examine the relationship between the LDL-C/HDL-C ratio and all-cause mortality. When the LDL-C/HDL-C ratio was used as a continuous variable, there was no significant relationship between the LDL-C/HDL-C ratio and all-cause mortality in any of the three models (all *P* > 0.05). Then, the LDL-C/HDL-C ratio was converted from a continuous variable to a categorical variable. The patients were divided into five groups according to the quintiles of the LDL-C/HDL-C ratio. Compared to the reference group (Q3: 1.67–2.10), patients in both the lower (Q1 and Q2: < 1.67) and higher (Q4 and Q5: ≥2.10) LDL-C/HDL-C ratio groups had higher mortality in all models (all *P* < 0.05). In model 2 (fully adjusted model), both the low LDL-C/HDL-C ratio and high LDL-C/HDL-C ratio groups had relatively higher mortality (< 1.67: HR 1.81, 95% CI: 1.08–3.03; ≥2.10: HR 2.00, 95% CI: 1.18–3.39). In addition, the regression coefficients (β) were added to the regression model (Table S3). These results suggest that the relationship between the LDL-C/HDL-C ratio and all-cause mortality is likely to be nonlinear. The fully adjusted smooth curve fitting showed a U-shaped relationship between the baseline LDL-C/HDL-C ratio and all-cause mortality (Fig. 1).

**Table 2:** Association between the LDL-C/HDL-C ratio and all-cause mortality during the follow-up period

LDL-C/HDL-C ratio	Events, %	Crude model		Model 1		Model 2	
		HR (95% CI) ^a^	*P* value	HR (95% CI)	*P* value	HR (95% CI)	*P* value
Continuous	157/6947 (2.29)	0.93 (0.73, 1.19)	0.558	1.05 (0.83, 1.34)	0.674	1.02 (0.76, 1.36)	0.912
Quintiles							
< 1.16	40/1387 (2.88)	2.12 (1.23, 3.67)	0.007	1.88 (1.09, 3.25)	0.024	1.98 (1.13, 3.46)	0.017
1.16–1.67	32/1389 (2.30)	1.69 (0.96, 2.98)	0.070	1.57 (0.89, 2.78)	0.118	1.64 (0.92, 2.90)	0.091
1.67–2.10	19/1389 (1.37)	Reference		Reference		Reference	
2.10–2.79	30/1387 (2.16)	1.59 (0.89, 2.82)	0.115	1.75 (0.98, 3.11)	0.057	1.80 (1.01, 3.22)	0.048
≥ 2.79	36/1389 (2.59)	1.91 (1.09, 3.33)	0.023	2.12 (1.21, 3.70)	0.008	2.24 (1.24, 4.03)	0.008
Categories							
< 1.67	72/2776 (2.59)	1.91 (1.15, 3.16)	0.012	1.73 (1.04, 2.87)	0.034	1.81 (1.08, 3.03)	0.024
1.67–2.10	19/1389 (1.37)	Reference		Reference		Reference	
≥ 2.10	66/2776 (2.38)	1.75 (1.05, 2.91)	0.032	1.93 (1.16, 3.23)	0.011	2.00 (1.18, 3.39)	0.010
*P* for trend		0.570		0.598		0.767	

^a^ Cox proportional hazards models were used to estimate hazard ratio (HR) and 95% confidence interval (95% CI)

Abbreviations: *HR* hazard ratio; *CI* confidence interval; *LDL-C* low density lipoprotein-cholesterol; *HDL-C* high-density lipoprotein cholesterol

Model 1: adjusted for none. Model 2: adjusted for age, sex. Model 3: adjuste d for age, sex, BMI, SBP, DBP, TG, Hcy, FBG, SUA, eGFR, smoking, alcohol consumption, diabetes, stroke, CVD and anti-hypertensive drugs

As shown in Table 3, stratified analyses were conducted with stratification by sex, BMI, stroke, CVD, diabetes, eGFR, smoking and alcohol consumption. The U-shaped association was consistently observed in all subgroups, and there was no significant interaction between the LDL-C/HDL-C ratio and all-cause mortality in any subgroup (all *P* for interaction > 0.05). The regression coefficients (β) were added to the subgroup analysis model (Table S4).

**Table 3:** The subgroup analysis for the LDL-C/HDL-C ratio on all-cause mortality

Subgroups	Categories of the LDL-C/HDL-C ratio, HR (95% CI)			*P* for interaction
	Low (< 1.67)	Reference (1.67–2.10)	High (≥2.10)	
Sex				0.249
male	1.38 (0.76, 2.50)	1	1.51 (0.80, 2.84)	
female	3.33 (1.15, 9.69)	1	3.92 (1.35, 11.36)	
BMI, kg/m^2^				0.99
< 24	1.81 (1.01, 3.26)	1	2.13 (1.14, 3.98)	
≥ 24	1.85 (0.62, 5.50)	1	1.73 (0.64, 4.70)	
Stroke				0.623
No	1.71 (1.00, 2.91)	1	1.81 (1.05, 3.14)	
Yes	3.63 (0.40, 32.93)	1	6.89 (0.79, 60.40)	
CVD				0.21
No	1.58 (0.93, 2.69)	1	2.01 (1.17, 3.45)	
Yes	4.23 (0.43, 41.60)	1	1.38 (0.10, 18.38)	
Diabetes				0.25
No	1.63 (0.95, 2.79)	1	1.68 (0.96, 2.94)	
Yes	6.38 (0.80, 50.66)	1	8.18 (1.04, 64.02)	
eGFR, ml/min/1.73m^2^				0.097
< 60	5.35 (1.24, 23.09)	1	8.93 (2.02, 39.51)	
≥ 60	1.45 (0.82, 2.55)	1	1.44 (0.80, 2.57)	
Smoking				0.452
No	2.10 (1.16, 3.80)	1	2.07 (1.12, 3.82)	
Yes	1.12 (0.40, 3.18)	1	1.86 (0.64, 5.37)	
Alcohol consumption				0.969
No	1.75 (1.01, 3.03)	1	1.99 (1.13, 3.48)	
Yes	1.87 (0.40, 8.68)	1	1.91 (0.38, 9.68)	

Adjusted for age, sex, BMI, SBP, DBP, TG, Hcy, FBG, SUA, eGFR, smoking, alcohol consumption, diabetes, stroke, CVD and anti-hypertensive drugs, if not be stratified

**Fig. 2:**
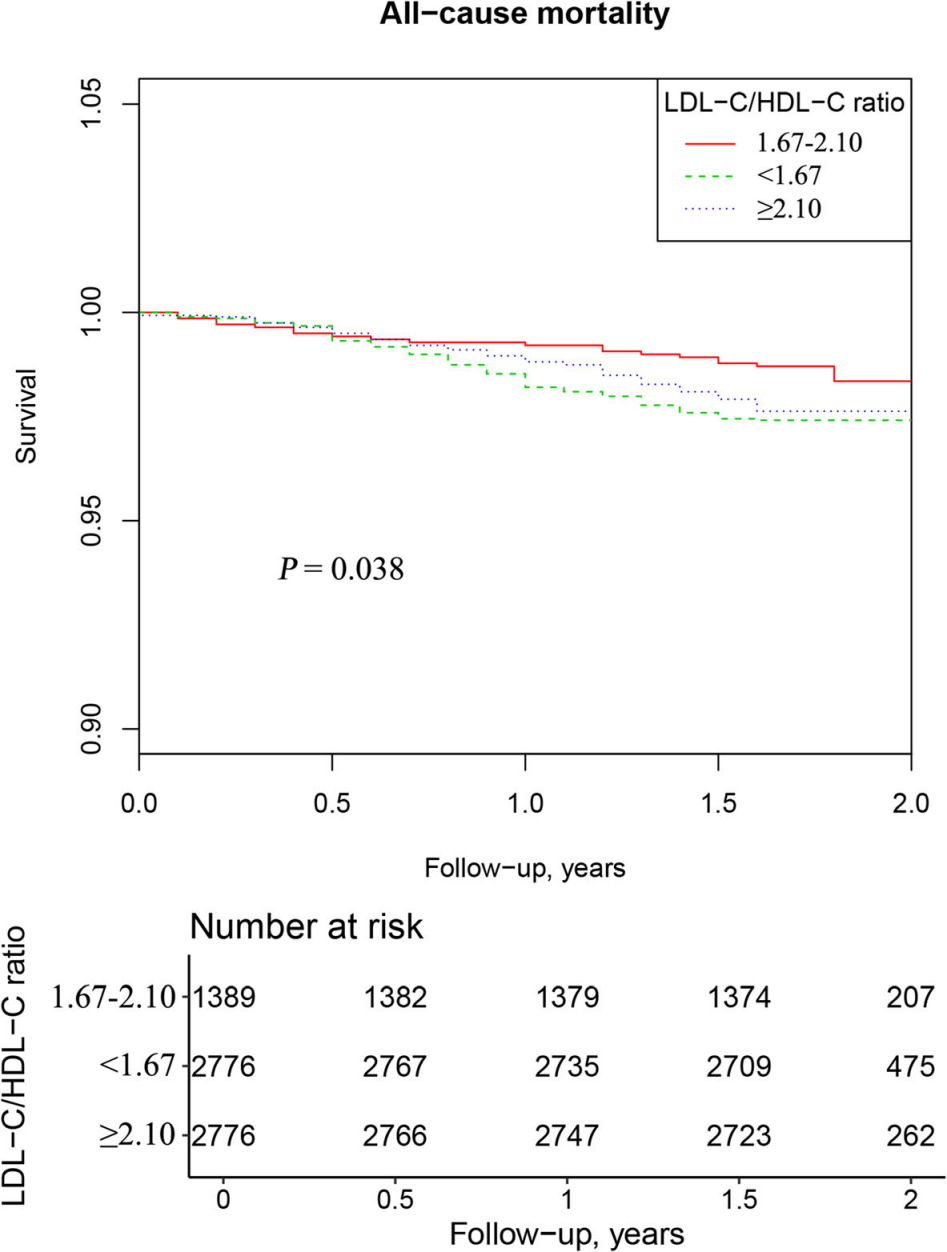
Kaplan–Meier survival curve estimates for all-cause mortality in the elderly hypertensive population

The Kaplan–Meier survival curve for all-cause mortality stratified by LDL-C/HDL-C ratio levels is shown in Fig. 2. Compared with the lower and higher LDL-C/HDL-C ratio groups, patients with LDL-C/HDL-C ratios of 1.67–2.10 had a significantly higher survival probability (log-rank *P* < 0.05).

## Discussion

In the current study, we found a U-shaped relationship between the LDL-C/HDL-C ratio and all-cause mortality in the elderly hypertensive population in China. Both lower and higher LDL-C/HDL-C ratios were associated with higher all-cause mortality. The optimal range of the LDL-C/HDL-C ratio was 1.67–2.10. Compared with the lower and higher LDL-C/HDL-C ratio groups, patients with LDL-C/HDL-C ratios of 1.67–2.10 had a significantly higher survival probability.

Although CVD is one of the leading causes of death in elderly patients with hypertension, studies on the relationship between the LDL-C/HDL-C ratio and all-cause mortality have been limited. Therefore, we also analyzed the LDL-C/HDL-C ratio and CVD, to thoroughly investigate the predictive value of the LDL-C/HDL-C ratio. Gragnano, F et al. [15] described the importance of lowering LDL-C to reduce the risk of CVD. However, studies have yielded inconsistent conclusions regarding the predictive value of the LDL-C/HDL-C ratio. Matsumoto I et al. [6] analyzed 687 patients who underwent PCI (mean age 67.7 ± 9.9 years, mean follow-up years = 2.75 years) and found a positive association between the LDL-C/HDL-C ratio and CVD. They suggested that the LDL-C/HDL-C ratio should be controlled below 1.5. Zhong et al. [7] enrolled 1937 acute coronary syndrome (ACS) patients (mean age 64.0 ± 10.8 years, mean follow-up =1.00 years), and found that a high LDL-C/HDL-C ratio was associated with an increased risk of CVD. They suggested that the LDL-C/HDL-C ratio should be controlled below 2.7. Yokokawa et al. [5] included 8714 male patients (mean age 63.7 ± 11.5 years, mean follow-up =2.7 ± 0.9 years) and found that compared with patients with an LDL-C/HDL-C ratio < 2.6, patients with a ratio > 2.6 had a higher risk of CVD. The findings of the above studies suggest that there may be a positive association between the LDL-C/HDL-C ratio and CVD, and different studies have proposed different reference ranges for the LDL-C/HDL-C ratio. In contrast, You et al. [9] included 356 patients with intracranial hemorrhage (mean age 64.1 ± 13.7 years, mean follow-up =0.22 years) and found that the LDL-C/HDL-C ratio was negatively correlated with all-cause mortality, they suggested that the LDL-C/HDL-C ratio should be controlled above 2.96. Liu et al. [10] recruited 3250 stroke patients (mean age 63.72 ± 11.33 years, mean follow-up =1.00 years) and found a negative relationship between the LDL-C/HDL-C ratio and all-cause mortality. Mortality was lowest when the LDL-C/HDL-C ratio was between 2.23 and 2.88. The findings of the above studies suggest that the relationship between the LDL-C/HDL-C ratio and all-cause mortality may be negative, and the proposed optimal range of the LDL-C/HDL-C ratio has been inconsistent. These conflicting results can be attributed to differences in the study populations, follow-up durations, and end-point events. Consequently, the exact relationship between the LDL-C/HDL-C ratio and all-cause mortality and the optimal LDL-C/HDL-C ratio are still unclear, which prompted us to conduct the current study.

The mechanism driving the association identified in this study remains unclear. Several possible mechanisms could explain the observed relationship. In this study, when the LDL-C/HDL-C ratio was low, there was a negative correlation between the LDL-C/HDL-C ratio and all-cause mortality. As shown in Table S2, we found that all deaths caused by respiratory diseases occurred in group of patients with LDL-C/HDL-C ratios < 1.67. A lower LDL-C/HDL-C ratio may be associated with inflammation, increasing the energy needed for respiration, which in turn would aggravate respiratory failure [16]. Notably, the lower LDL-C/HDL-C ratio was caused by higher HDL-C levels [17]. Consistent with our findings, Bowe et al. [18] included 1,764,986 US adults (mean follow-up =9.1 years) and found a U-shaped relationship between HDL-C and all-cause mortality, suggesting that a higher HDL-C level was associated with increased mortality. C.M. Madsen et al. [19] included 116,508 patients (mean follow-up =6.0 years) and found that higher HDL-C levels were associated with increased all-cause mortality. It might be that a higher HDL-C level is associated with genetic variability, including mutations in ABCA1, LIPC, and SCARB1, which in turn promote the occurrence and progression of CVD [20, 21]. However, HDL-C lost its protective effect at a higher level, paradoxically enhancing senescence and impairing endothelial progenitor cell vascularization and angiogenesis [22, 23]. These mechanisms suggest that the conformational and functional properties of HDL particles may change when the level of HDL-C is high, which may lead to harmful effects. When the LDL-C/HDL-C ratio was high, it was positively associated with all-cause mortality. A higher LDL-C/HDL-C ratio may promote coronary inflammation [24], and may increase the vulnerability of coronary plaques to rupture [24, 25]. In addition, a higher LDL-C/HDL-C ratio has been found to be associated with increased LDL-C levels [17]. Higher LDL-C levels have also been shown to be associated with increased mortality, mainly through mechanisms involving oxidative stress and the inflammatory response [26, 27]. Admittedly, all-cause mortality involves a variety of factors, so it is difficult to fully explain the mechanism underlying the relationship between the LDL-C/HDL-C ratio and all-cause mortality. Therefore, further basic experiments are needed to fully elucidate the specific biological mechanism underlying this connection.

Compared with the normal population, elderly hypertensive patients have higher mortality [28]. Therefore, it is more important to identify a valuable predictor of mortality in this population. The LDL-C/HDL-C ratio might be a valuable indicator that could be used for prognostic prediction because it simultaneously reflects the levels of both LDL-C and HDL-C. More importantly, a U-shaped relationship has a greater clinical significance than a linear relationship [29], because it suggests that there might be a target optimal range for the LDL-C/HDL-C ratio.

### Study strengths and limitations

This cohort study found a U-shaped relationship between the LDL-C/HDL-C ratio and all-cause mortality in elderly people with hypertension in a large sample population. Our findings provide a new therapeutic target for lipid-lowering therapy in elderly patients with hypertension. Nevertheless, some limitations should be noted. First, the present study did not analyze cause-specific mortality due to the small sample sizes in the subgroups. However, this study is still ongoing, so the number of deaths will continue to increase. Second, our study population was elderly patients with hypertension, so the generality of our conclusions is limited. Third, we only analyzed the LDL-C/HDL-C ratio at baseline and did not observe dynamic changes over time. Finally, most of the evidence regarding the LDL-C/HDL-C ratio has come from studies performed in Asian populations, and large prospective cohort studies are needed to examine the predictive value of the LDL-C/HDL-C ratio for CVD or all-cause mortality in non-Asian populations.

## Conclusions

The present study showed a U-shaped relationship between the LDL-C/HDL-C ratio and all-cause mortality in elderly hypertensive patients in China. Both lower and higher LDL-C/HDL-C ratios were associated with higher all-cause mortality. Compared with the lower and higher LDL-C/HDL-C ratio groups, patients with LDL-C/HDL-C ratios of 1.67–2.10 had a significantly higher survival probability. These results suggest that the LDL-C/HDL-C ratio can be a valuable predictor of all-cause mortality in elderly hypertensive patients. For elderly patients with hypertension, the LDL-C/HDL-C ratio may be one of the targets of lipid-lowering therapy. Further large prospective studies are needed to confirm these findings and elucidate their clinical implications.

## Supplementary Information

**Additional file 1: Table S1.** The study inclusion and exclusion criteria. **Table S2.** Mortality during follow-up was described according to classification of LDL-C/HDL-C ratio. **Table S3.** Association between the LDL-C/HDL-C ratio and all-cause mortality during the follow-up period. **Table S4.** The subgroup analysis for LDL-C/HDL-C ratio on all-cause mortality.

## Data Availability

The datasets generated and analyzed during the current study are not publicly available because this study is still on-going and the follow-up is not finished, but are available from the corresponding author on reasonable request.

## Abbreviations

BMI: Body mass index
CVD: Cardiovascular disease
TC: Total cholesterol
TG: Triglyceride
LDL-C: Low density lipoprotein cholesterol
HDL-C: High density lipoprotein cholesterol
SUA: Serum uric acid
eGFR: Estimated glomerular filtration rate
Hcy: Homocysteine
FBG: Fasting blood glucose
HR: Hazard ratio
CI: Confidence interval
