# Supplementary material for: A U-shaped association between the LDL-cholesterol to HDL-cholesterol ratio and all-cause mortality in elderly hypertensive patients: a prospective cohort study
Source: Lipids Health Dis. 2020 Nov 12;19:238. doi: 10.1186/s12944-020-01413-5 (PMC7659118; doi:10.1186/s12944-020-01413-5)
Supplement: Supplementary file 1 — Additional file 1: Table S1. The study inclusion and exclusion criteria. Table S2. Mortality during follow-up was described according to classification of LDL-C/HDL-C ratio. Table S3. Association between the LDL-C/HDL-C ratio and all-cause mortality during the follow-up period. Table S4. The subgroup analysis for LDL-C/HDL-C ratio on all-cause mortality. [file 12944_2020_1413_MOESM1_ESM.docx]

| Table S1. The study inclusion and exclusion criteria |
| --- |
| Inclusion criteria |
| 1. 18 years of age or older |
| 1. hypertension defined as office systolic blood pressure (SBP) values ≥140 mmHg and/or diastolic BP (DBP) values ≥90 mmHg, self-reported history of hypertension, or the use of antihypertensive drug(s) at baseline |
| 1. signed informed consent |
| Exclusion criteria |
| 1. psychological or nervous system impairment resulting in an inability to demonstrate informed consent |
| 1. unable to be followed up according to the study protocol, or plans to relocate in the near future |
| 1. patients who were not suitable for inclusion or for long-term follow-up as assessed by the study physicians |

| Table S2. Mortality during follow-up was described according to classification of LDL-C/HDL-C ratio | | | | |
| --- | --- | --- | --- | --- |
| LDL-C/HDL-C ratio* | <1.67 | 1.67-2.10 | ≥2.10 | *P*-value |
| Number | 2,776 | 1,389 | 2,776 |  |
| Male, n (%) | 1,527 (55.01) | 629 (45.28) | 1,188 (42.80) | <0.001 |
| Age, years | 71.71 ± 5.55 | 71.10 ± 5.36 | 70.73 ± 4.97 | <0.001 |
| BMI, kg/m2 | 21.38 ± 4.22 | 22.77 ± 3.07 | 23.85 ± 3.23 | <0.001 |
| All-cause mortality, n (%) | 72 (2.59) | 19 (1.37) | 66 (2.38) | 0.037 |
| Causes of death, n (%) |  |  |  | 0.047 |
| Stroke | 19 (26.39) | 3 (15.79) | 21 (31.82) |  |
| CVD | 16 (22.22) | 4 (21.05) | 20 (30.30) |  |
| Cancer | 17 (23.61) | 9 (47.37) | 11 (16.67) |  |
| Respiratory disease | 6 (8.33) | 0 (0.00) | 0 (0.00) |  |
| Others | 14 (19.44) | 3 (15.79) | 14 (21.21) |  |

Abbreviations: BMI, body mass index; CVD, cardiovascular diseases; LDL-C, low density lipoprotein cholesterol; HDL-C, high density lipoprotein cholesterol.

*Data are presented as number (%) or mean ± standard deviation.

| Table S3. Association between the LDL-C/HDL-C ratio and all-cause mortality during the follow-up period | | | | | | | |
| --- | --- | --- | --- | --- | --- | --- | --- |
| LDL-C/HDL-C ratio | Events, % | Crude model |  | Model 1 |  | Model 2 |  |
|  |  | β (95% CI) | *P* value | β (95% CI) | *P* value | β (95% CI) | *P* value |
| Continuous | 157/6,947 (2.29) | -0.08 (-0.32, 0.17) | 0.551 | 0.06 (-0.19, 0.30) | 0.658 | 0.02 (-0.28, 0.32) | 0.906 |
| Quintiles |  |  |  |  |  |  |  |
| <1.16 | 40/1,387 (2.88) | 0.76 (0.21, 1.31) | 0.007 | 0.64 (0.08, 1.19) | 0.025 | 0.68 (0.11, 1.26) | 0.020 |
| 1.16-1.67 | 32/1,389 (2.30) | 0.53 (-0.04, 1.10) | 0.069 | 0.45 (-0.13, 1.03) | 0.125 | 0.49 (-0.09, 1.08) | 0.099 |
| 1.67-2.10 | 19/1,389 (1.37) | Reference |  | Reference |  | Reference |  |
| 2.10-2.79 | 30/1,387 (2.16) | 0.47 (-0.11, 1.05) | 0.115 | 0.57 (-0.02, 1.15) | 0.057 | 0.60 (0.01, 1.20) | 0.048 |
| ≥2.79 | 36/1,389 (2.59) | 0.65 (0.09, 1.21) | 0.023 | 0.76 (0.20, 1.33) | 0.008 | 0.81 (0.20, 1.41) | 0.009 |
| Categories |  |  |  |  |  |  |  |
| <1.67 | 72/2,776 (2.59) | 0.65 (0.14, 1.16) | 0.012 | 0.55 (0.04, 1.06) | 0.036 | 0.59 (0.07, 1.12) | 0.027 |
| 1.67-2.10 | 19/1,389 (1.37) | Reference |  | Reference |  | Reference |  |
| ≥2.10 | 66/2,776 (2.38) | 0.56 (0.05, 1.08) | 0.032 | 0.67 (0.15, 1.19) | 0.012 | 0.70 (0.16, 1.24) | 0.011 |
| *P* for trend |  | 0.566 | | 0.575 | | 0.750 | |

Abbreviations: CI, confidence interval; LDL-C, low density lipoprotein-cholesterol; HDL-C, high-density lipoprotein cholesterol.

Model 1: adjusted for none. Model 2: adjusted for age, sex. Model 3: adjusted for age, sex, BMI, SBP, DBP, TG, Hcy, FBG, SUA, eGFR, smoking, alcohol consumption, diabetes, stroke, CVD and anti-hypertensive drugs.

| Table S4. The subgroup analysis for LDL-C/HDL-C ratio on all-cause mortality | | | | |
| --- | --- | --- | --- | --- |
| Subgroups | Categories of LDL-C/HDL-C ratio, β (95% CI) | |  | *P* for interaction |
|  | Low (<1.67) | Reference (1.67-2.10) | High (≥2.10) |  |
| Sex |  |  |  | 0.239 |
| male | 0.30 (-0.31, 0.91) | Ref | 0.41 (-0.24, 1.06) |  |
| female | 1.23 (0.16, 2.31) | Ref | 1.37 (0.30, 2.45) |  |
| BMI, kg/m^2^ |  |  |  | 0.994 |
| <24 | 0.58 (-0.02, 1.19) | Ref | 0.77 (0.13, 1.41) |  |
| ≥24 | 0.63 (-0.49, 1.74) | Ref | 0.54 (-0.47, 1.56) |  |
| Stroke |  |  |  | 0.610 |
| No | 0.54 (-0.01, 1.08) | Ref | 0.60 (0.04, 1.16) |  |
| Yes | 1.34 (-0.93, 3.62) | Ref | 2.02 (-0.22, 4.26) |  |
| CVD |  |  |  | 0.232 |
| No | 0.46 (-0.09, 1.00) | Ref | 0.71 (0.15, 1.26) |  |
| Yes | 1.42 (-0.94, 3.77) | Ref | 0.22 (-2.48, 2.92) |  |
| Diabetes |  |  |  | 0.248 |
| No | 0.48 (-0.07, 1.03) | Ref | 0.53 (-0.05, 1.10) |  |
| Yes | 1.92 (-0.20, 4.03) | Ref | 2.16 (0.06, 4.26) |  |
| eGFR, ml/min/1.73m^2^ |  |  |  | 0.093 |
| <60 | 1.74 (0.23, 3.24) | Ref | 2.29 (0.76, 3.83) |  |
| ≥60 | 0.37 (-0.21, 0.94) | Ref | 0.37 (-0.23, 0.96) |  |
| Smoking |  |  |  | 0.460 |
| No | 0.75 (0.15, 1.36) | Ref | 0.74 (0.12, 1.37) |  |
| Yes | 0.08 (-0.98, 1.14) | Ref | 0.60 (-0.49, 1.68) |  |
| Alcohol consumption |  |  |  | 0.959 |
| No | 0.57 (0.01, 1.13) | Ref | 0.70 (0.12, 1.27) |  |
| Yes | 0.58 (-0.97, 2.13) | Ref | 0.72 (-0.93, 2.36) |  |

Adjusted for age, sex, BMI, SBP, DBP, TG, Hcy, FBG, SUA, eGFR, smoking, alcohol consumption, diabetes, stroke, CVD and anti-hypertensive drugs, if not be stratified.
